# Enzymatically hydrolyzed diet improves growth performance and intestinal microbiome in growing pigs

**DOI:** 10.3389/fnut.2024.1485017

**Published:** 2024-12-13

**Authors:** Tianye Gong, Mengting Ji, Yuting Yang, Jingchao Liu, Yuxuan Gong, Sijun Liu, Yan Zhao, Guoqing Cao, Xiaohong Guo, Yang Yang, Bugao Li

**Affiliations:** ^1^College of Animal Science, Shanxi Agricultural University, Taigu, Shanxi, China; ^2^Key Laboratory of Farm Animal Genetic Resources Exploration and Breeding of Shanxi Province, Taigu, China

**Keywords:** enzymatically hydrolyzed diet, growing pig, growth performance, digestion, fecal microbiota

## Abstract

**Background:**

The use of enzymes within pig feed can reduce the challenges associated with antibiotic-free animal feeding. However, this enzymatic effect is often limited by the internal and external gut environment. This study aimed to improve diet quality and assess the impact of an enzymatically hydrolyzed diet (EHD) on growth performance, meat quality, and intestinal health in growing pigs.

**Methods:**

The EHD was prepared by treating a liquid basal diet with a compound enzyme preparation (5 non-starch polysaccharides (NSP) enzymes: cellulase, pectinase, xylanase, *β*- glucanase, *α*-galactosidase; 3 exogenous digestive enzymes: amylase, lipase, protease; lysozyme, and glucose oxidase) in a 1:2.5 ratio with water and heated at 40°C for 1 h. Thirty-six growing pigs (average body weight 25 ± 0.5 kg; age 75 ± 3 days) from the Duroc × Landrace × Yorkshire crossbreed were randomly divided into three dietary groups: solid basal diet (S-CON), liquid basal diet (L-CON), and EHD.

**Results:**

Enzymatic pre-treatment reduced the anti-nutritional factors (ANFs) in the diets (*p* < 0.01). Additionally, the gluconic acid in the EHD might reduce the pH of diets and inhibit pathogenic bacteria growth. Pigs fed the EHD had higher average daily gains (*p* < 0.01) and lower feed-to-gain ratios (*p* < 0.01). Muscle samples revealed higher meat redness and reductions in drip loss, shear force, cooking loss, and meat yellowness (*p* < 0.01). Moreover, the EHD increased intestinal concentrations of amylase and cellulase (*p* < 0.01). In terms of gut health, pigs on the EHD diet presented more aligned small intestinal villi, with improved villus height and villus crypt ratio (*p* < 0.01). There was also up-regulation of the abundance of the tight junction proteins *Occludin* and *ZO-1* and down-regulation of the mRNA expression of *TNF-α* and *IL-6* in the colon (*p* < 0.05). Additionally, the abundance of beneficial intestinal flora, particularly *Firmicutes* and *Lactobacillus*, increased significantly. *Lactobacillus* and *Prevotella* were positively correlated with increased short-chain fatty acids.

**Conclusion:**

Overall, the EHD substantially improved growth performance and intestinal health in pigs, providing a potential reference for improving the effectiveness of enzymatic pretreatment in animal diets.

## 1 Introduction

Given the growing concern for antibiotic-free animal food safety, enzyme additives have become a widely accepted and mature biotechnology for improving feed utilization (1). These additives also have the potential to improve the intestinal health and production performance of animals (2). Various studies indicate that enzymes with specific functions affect piglet nutrient metabolism differently (3). Glucose oxidase, in particular, shows promise as an antibiotic alternative in feed due to its antioxidant properties (4). Further, acid protease and neutral protease significantly increase the apparent metabolic rate of energy and crude protein (5). However, the effectiveness of enzyme additives can be inconsistent because enzymes, as macromolecular proteins, can lead to physiological limitations (6), such as enzyme pH sensitivity and varying residence times in the gastrointestinal tract (2, 7).

Corn and soybean meal are commonly used, high-quality protein and energy sources in swine feed. However, despite their benefits, they contain multiple ANFs that limit the feed bioavailability and impede intestinal absorption, potentially inhibiting the healthy growth and development of piglets (8–10). For example, corn contains high levels of the ANFs cellulose and xylan (11), these insoluble NSP inhibit the complete extraction of energy from the corn by the intestinal digestive juices, and pig intestines do not secrete the branched-chain amylase enzyme necessary to digest corn (12).

Pre-digested enzymatic treatment of feed shows promise as a nutritional strategy to address the physiological limitations of enzyme additives. Pre-treatment allows enzymes to adequately degrade ANFs under controlled time and temperature conditions, reducing the impact of the intestinal environment on enzyme activity (13, 14). Most of the contemporary literature focuses on the enzymatic pre-digestion of raw materials such as soybean meal, with the corresponding hydrolytic enzymes being primarily proteases and carbohydrases (15). However, there is limited research on the pre-digestion of complete diets, and few studies examine the combined use of multiple functional enzymes, including NSP enzymes, exogenous digestive enzymes, and growth-promoting glucose oxidase.

NSP enzymes can effectively degrade NSP in feed, by adding NSP enzymes, it can reduce the viscosity of digestive matter and promote the release and absorption of nutrients, so as to improve the nutritional value of feed (16). It has been found that the addition of NSP enzymes to feed can significantly increase the daily weight gain and feed conversion ratio of pigs. Moreover, the use of NSP enzymes also contributes to the improvement of the intestinal internal environment and immunity of pigs (17). The main purpose of exogenous digestive enzymes in swine feed is to increase the nutritional value and digestibility of feed, thus improving the growth performance and health of pigs (18). In the case of lysozyme and glucose oxidase, they have shown significant effects as feed additives in improving growth performance, intestinal health and immunity in pigs (19, 20).

This study aims to identify a complex enzyme preparation suitable for corn-soybean meal diets and to evaluate the resulting enzyme pre-digested diet (EHD) by evaluating its effectiveness, as well as its impact on growth performance, meat quality, digestive metabolism, intestinal barrier function, and fecal flora of growing pigs.

## 2 Materials and methods

### 2.1 Ethics statement

The animal experimental procedures were approved by the Animal Care and Use Committee of Shanxi Agricultural University (SXAU-EAW-2021MS.P.052801).

### 2.2 Materials

The equations should be inserted in editable format from the equation editor. Forage enzyme preparations (temperature range and enzyme activity detailed in Table 1) were purchased from SUNSON Biotechnology Co., Ltd. (Cangzhou, China). Standard gluconic acid was purchased from Solarbio (Beijing, China). Total amino acid assay kits (A026-1-1) were purchased by Jiancheng Bioengineering Technology (Nanjing, China). ELISA kits for detecting glycinin, *α*-conglycinin, *β*-conglycinin, and soybean trypsin inhibitor (STI) content were purchased from Jiangsu Meimian Industrial Co., Ltd. (Jiangsu, China). All other chemicals and solvents used were of analytical grade.

**Table 1:** Characteristics of forage enzyme preparation.

Item	Temperature range, °C	Enzyme activity, U/g
Amylase	30–65	≥ 1,500
Lipase	30–60	1,000
Protease	50–70	100
lysozyme	30–55	20
Glucose oxidase	30–60	100
Cellulase	40–60	100
Pectinase	40–60	250
Xylanase	35–60	100
β-Dextranase	40–60	500
α-Galactosidase	40–60	80

### 2.3 Preparation of enzymatically hydrolyzed diet

The basal diet was formulated according to the nutritional requirements for swine as recommended by the National Research Council (21). The ingredients and nutritional values are shown in Table 2. The substrates for the EHD group consisted of the solid basal diet (S-CON). These substrates were mixed with water in a ratio of 1:2.5 and treated with compound enzyme preparations. The liquid control group (L-CON) was prepared by adding the same amount of water without these enzymes. The substrates were then pre-digested at temperatures of 40, 50, and 60°C for 1 h, respectively. Each trial was performed in triplicate.

**Table 2:** Ingredient and nutrient levels of experiment basal diet (%, as-fed basis).

Item	Compositions, %	Item	Nutrient level^2^, %
Corn	61.73	CP	18.00
Extruded soybean	20.87	EE	15.56
Soybean meal	11.02	NDF	11.19
*L*-Lys HCl	0.50	ADF	4.43
*dL*-Met	0.22	Calcium	0.72
*L*-Threonine	0.20	Phosphorus	0.52
*L*-Tryptophan	0.08	SIDLys	1.32
Limestone	0.34	SIDMet	0.49
CaHPO_4_	2.07	SIDThr	0.86
NaCl	0.67	SIDTrp	0.27
Acidifier	0.30		
Premix^1^	2.00		
Total	100.00		

CP, crude protein; EE, ether extract; NDF, neutral detergent fiber; ADF, acid detergent fiber; SID, standardized ileal digestible.

1 The premix provided the following per kilogram of diet: vitamin A, 9,000 IU; vitamin B_1_, 5 mg; vitamin B_2_, 9 mg; vitamin B_6_, 32 mg; vitamin B_12_, 0.030 mg; vitamin D_3_, 1,900 IU; vitamin E, 22 IU; vitamin K, 35 mg; biotin 0.09 mg; calcium pantothenate, 15 mg; niacin, 29 mg; Cu, 21 mg; Fe, 65 mg; Mn, 45 mg; Zn, 65 mg; I, 0.55 mg; Se, 0.32 mg.

2 CP, EE, NDF, ADF, Calcium and Phosphorus are measured values (*n* = 3).

### 2.4 Chemical analysis and microscopic observation of diets

Liquid samples were collected to evaluate the bacteriostatic effect, and the remaining sample material was dried at 65°C for 48 h, cooled, ground, and prepared for conventional nutrient analysis. The dried samples were analyzed for organic matter (OM), crude protein (CP), ether extract (EE), calcium, and phosphorus according to AOAC methods (22). Dietary nitrogen content was measured before and after the pre-digestion treatment using a Kjeldahl automated apparatus (K9805, Shanghai Analytical Instrument Co., Ltd., Shanghai, China). The nitrogen content was then multiplied by a factor of 6.25 to estimate CP quantity. EE was measured by Soxhlet extraction (23). Neutral detergent fiber (NDF) and acid detergent fiber (ADF) were measured using a fiber analyzer (ANKOM A200i Fiber Analyzer, United States) following the method of McRoberts and Cherney (24). Calcium and phosphorus contents were measured using the method described by Hanson (25). Reducing sugar was quantified by the dinitro salicylic acid (DNS) method (26).

Enzymatic hydrolysis products in the supernatants were analyzed using a high-performance liquid chromatography system (HPLC) equipped with a reverse-phase column [Luna 3.5 μm C18(2) 100 Å, LC Column 250 mm × 4.6 mm, Phenomenex, Torrance, CA]. The mobile phase, consisting of methanol (65%) and water (35%), was pumped at a flow rate of 1.0 mL/min, and gluconic acid was detected at wavelengths of 325, 340, and 385 nm. Gluconic acid was used as an external standard. Physical property changes in diets before and after enzymatic pre-digestion were examined using scanning electron microscopy (KYKY-EM3200, China) and laser confocal microscopy. The relative fluorescence intensity on the surface of the substances was measured by rhodamine B staining (Solarbio, Beijing, China).

The bacteriostasis of the predigested feed was tested by bacterial culture. Set up test groups: negative control (CON): single colony + sterile water, experimental group (EHD): single colony + enzymatic solution. Take out the preserved bacteria of Escherichia coli and Salmonella from the refrigerator at −80°C, coat the activated bacteria in LB solid medium, put them in 37°C constant temperature incubators for 24 h, pick single colonies and inoculate them in LB liquid medium, incubate them at 180 r/min with shaking, and take samples for measuring their OD value (600 nm) every 15 min to draw the growth curve.

### 2.5 Feeding-experiment design

Thirty-six Duroc × Landrace × Yorkshire pigs (body weight (BW) = 25 ± 0.5 kg; age = 75 ± 3 days) were randomly assigned to three dietary treatment groups. Each group had three replicates with four pigs (two males and two females). The groups were as follows: the solid control (S-CON) group, which received the basal diet; the L-CON group, which received the S-CON mixed with water at a ratio of 1:2.5; and the EHD group, which received a basal diet mixed with water in the same ratio and supplemented with 4% forage enzyme preparations. Pigs were fed *ad libitum* twice daily at 8:00 and 17:00 during both the 7-day pretrial period and the 30-day trial period. Deworming, castration, and immunization were performed according to standard farm protocols prior to the commencement of the experiment.

### 2.6 Determination of nutrient digestibility

The metabolism test used metabolic cages and the total feces collection method to observe the effects on the apparent metabolic rate of nutrients and digestive enzyme activity in pigs. The cage dimensions were: length 1,200 mm, width 600 mm, height 800 mm. On day 20, three healthy pigs with similar weights were selected from each group. The entire phase included a 5-day pretrial period followed by a 5-day fecal collection period (27). Daily intake was set at 4% of body weight, fed twice daily at 8:00 and 17:00, with water provided *ad libitum*. Fresh feces were accurately collected without contamination over the 5-day period. Fecal samples were treated with 10% sulfuric acid (H₂SO₄), stored at −20°C, then dried, cooled, ground, and prepared for later chemical analyses.

The contents of OM, CP, EE, NDF, and ADF in feces were determined using the same methods applied to the diet analysis as described above. Nutrient digestibility was assessed using the total feces collection method as previously described (25). Intestinal digesta and mucosa was homogenized in cold saline and the supernatant was extracted after centrifugation at 2,500 r/min for 10 min, to prepare for the digestive enzyme activity assay. The activity of four enzymes was determined by colorimetry using commercial kits (Jiancheng, Nanjing, China)-*α*-amylase (starch-iodine colorimetry), lipase (colorimetry), cellulase (colorimetry), and trypsin (UV colorimetry).

### 2.7 Samples collection

Feed intake and refusal were recorded daily. Pigs were weighed after fasting for 12 h at the beginning and end of the experiment. At the conclusion of the trial, three pigs from each treatment group, close to the group’s average weight, were selected. Blood samples were collected from these pigs via the jugular vein into heparinized collection tubes. About 10 mL of blood was collected from the BD Serum Collection Tube and dispensed into red biochemical tubes. Immediately after blood collection, the blood was placed in an ice box at a low temperature for 15–20 min, and then centrifuged at 3,000–4,000 rpm for 5 min at 4°C, and then the supernatant was dispensed into sterile centrifugal tubes and then frozen in a refrigerator at −20°C for spare use. Subsequently, the pigs were euthanized and slaughtered using standard humane procedures.

### 2.8 Meat quality analysis

Meat quality indicators, including pH, meat color, Warner-Bratzler shear force (WBSF), and drip loss, were measured. The pH of the longissimus dorsi (LD) muscle, located between the last and penultimate thoracic vertebrae, was measured at 45 min and 24 h post-slaughter using a pH meter (pH-STAR, SFK-Technology, Denmark). Meat color was measured directly on the LD muscle at the thoracolumbar vertebral junction using a spectrophotometric colorimeter (Konica Minolta Inc., Tokyo, Japan) within 1 to 2 h post-slaughter. Drip loss was determined using the dropper method on LD muscle samples taken from the third and fourth thoracic vertebrae within 1 to 2 h post-slaughter. Trimmed muscle cubes (3 cm^3^) were weighed, held at a constant temperature of 4°C for 48 h, then reweighed. To measure WBSF, LD muscles from the 13th to 16th lumbar vertebrae were refrigerated at 4°C for 72 h to mature, then heated in a thermostatic water bath until reaching an internal temperature of 70°C. After cooling to room temperature, cylindrical meat samples (3 cm in length) were prepared by shearing parallel to the muscle fibers.

### 2.9 Intestinal morphology and histology

Intestinal samples were collected immediately post-slaughter and kept on ice boards. The intestinal contents were manually flushed out with saline, and approximately 2 cm sections of duodenum, jejunum, and ileum were fixed in 4% paraformaldehyde. Cross-sectional slices of each specimen were prepared, subjected to hematoxylin and eosin (H&E) staining, and subsequently encased in a neutral resin for preservation. The intestinal morphology was observed using the Leica DMi8 Microsystem and Leica Application Suite 3.7.0 (Leica, Wetzlar, Germany). Three slices were selected from each cross-section of the intestinal canal, and five intact and well-oriented villus-crypt units were randomly chosen from each slice. Villus height (VH) and crypt depth (CD) were measured using ImageJ software (Image-Pro Plus 6.0, Media Cybernetics, United States), then the villus height/crypt depth (V/C) was calculated.

### 2.10 Western blotting and quantitative real-time PCR

Total protein (TP) extracts from scraped colon mucosa were obtained using a Total Protein Extraction Kit (KeyGen BioTECH, Nanjing, China). Equal amounts of protein were separated by sulfate-polyacrylamide gel electrophoresis and transferred to polyvinylidene fluoride membranes. The membranes were then blocked with 5% skim milk (Sangon Biotech Co., Ltd., Shanghai, China) and then incubated with primary antibodies overnight at 4°C. Subsequently, they were incubated with the corresponding secondary antibodies at room temperature for 1 h. Antibodies used included those against Occludin, Zonula Occludens-1 (ZO-1), and *β*-actin. Western blot analysis was performed using an Odyssey infrared imaging system (LI-COR Biosciences, Lincoln, NE, United States).

Total RNA was extracted from pig colon tissue using Trizol reagent (Sigma, Saint Louis, MO), and cDNA was synthesized with a reverse transcription kit (TransGen, Beijing, China). qRT-PCR was used to measure the relative mRNA expression of tight junction (TJ) proteins (*Occludin* and *ZO-1*) and inflammatory cytokines (Interleukin 4, Interleukin 6; Interleukin 10, and tumor necrosis factor) in the colon. qRT-PCR was conducted using a CFX RT-PCR detection system (BioRad, Hercules, CA, United States) and a SYBR Green RT-PCR kit (TAKARA Co., Ltd) with the following cycle parameters: 95°C for 10 min, followed by 45 two-step cycles of 95°C for 7 s and 60°C for 34 s, and a cooling step at 40°C for 60 s. Primer sequences are listed in Table 3. Relative mRNA content was standardized to *β-actin*, and gene expression changes were calculated using the 2^−ΔΔCt^ method.

**Table 3:** Primer sequences for real-time PCR^1^.

Gene^2^	Sequence (5′ → 3′)^3^	Product size, bp	Accession number
*Occludin*	F: CGAGACAGACTACACGACGG R: TTCATCAGCAGCAGCCATGT	247	NM_001163647.2
*ZO-1*	F: AGCCCGAGGCGTGTTT R: GGTGGGAGGATGCTGTTG	147	XM_021098856.1
*IL-4*	F: TCACCTCCCAACTGATCCCA R: GCTCCATGCACGAGTTCTTT	144	NM_214123.1
*IL-6*	F:AGACCCTGAGGCAAAAGGGAAA R: CGGCATCAATCTCAGGTGCC	209	NM_214399.1
*IL-10*	F: CCACAAGTCCGACTCAACGA R: GGCAACCCAGGTAACCCTTA	267	NM_214041.1
*TNF-α*	F: TGCACTTCGAGGTTATCGGC R: CGGCTTTGACATTGGCTACAA	141	NM_214022.1
*β-actin*	F: CGGCTTTCGGTTGAGCTGAC R: GCCGTACCCACCAGAGTGAA	159	XM_021086047.1

1 Primers designed using Primer Express software (Sangon Biotech, Shanghai, China).

2 *ZO-1, zonula occludens 1; IL-4, interleukin 4; IL-6, interleukin 6; IL-10, interleukin 10; TNF-α, tumor necrosis factor alpha.*

3 F, forward; R, reverse.

### 2.11 Chemical analyses of short-chain fatty acids

Colonic contents were cooled in liquid nitrogen for 24 h then stored at −80°C. The detection of SCFA followed the method of Liu et al. (28). Briefly, colon content samples were centrifuged in ultrapure water to obtain the supernatant, which was then mixed with crotonic acid metaphosphate and stored at −20°C. Before measurement, the solution was thawed, filtered through a 0.22 μm aqueous membrane, centrifuged, and the supernatant was injected into a gas chromatograph (TRACE 1300 Gas Chromatograph Mass Spectrometer, Thermo Scientific, United States). The chromatographic conditions were as follows: injection volume 2.0 μL, injection temperature of 220°C, split ratio 6, constant flow rate of 0.8 mL/min, initial column temperature of 70°C, and detector temperature of 220°C. Gas flow rates were set to hydrogen 35 mL/min, air 350 mL/min, and tail blow 40 mL/min.

### 2.12 Fecal microbiological analysis

On 30th day, fecal samples were collected from the anal orifice morning after feeding. These samples were cooled in liquid nitrogen for 24 h and then stored at −80°C. They were sent to the Majorbio sequencing platform (Shanghai, China) for 16S rRNA sequencing and data analysis. The fecal samples were used for total microbial DNA extraction using the TIAN amp Bacteria DNA kit (Tiangen Biotech Inc., Beijing, China). The final DNA concentration and purification were determined using the NanoDrop 2000 UV–Vis spectrophotometer and DNA quality was checked by 1% agarose gel electrophoresis (Thermo Fisher Scientific, Waltham, Massachusetts, United States). Primers 341F (5’-CCTAYGGGRBGCASCAG-3′) and 806R (5’-GGACTACNNGGGTATCTAAT-3′) were used to amplify the V3-V4 variable regions of the 16S rRNA gene through PCR the thermocycler PCR system (Thermo Fisher Scientific, Waltham, Massachusetts, United States). The amplified products were purified with AxyPrep DNA Gel Extraction Kit (Axygen Biosciences, Union City, California, United States) and quantified with QuantiFluor™-ST (Promega, United States) according to the manufacturer’s protocol. Purified amplicons were pooled in equimolar and paired-end sequenced (2 × 300 bp) on an Illumina MiSeq platform (Illumina, San Diego, United States). All the raw data were filtered, denoised, merged, and non-chimeric by Qiime2 DADA2 plug-in to form ASVs. Classification of ASVs on species based on sklearn algorithm. The alpha and beta diversity of fecal microorganisms were analyzed using four indices: Chao, Shannon, Simpson, and Faith. Characteristic genera unique and common to each sample subgroup were identified and calculated to find significant biological markers, which were then illustrated in linear discriminant analysis effect size (LEfSe) maps. The comparative analysis of correlations between fecal microflora abundance and SCFAs was completed using the Wekemo Bioincloud, a dynamic real-time interactive online platform for data analysis.^1^

### 2.13 Statistical analysis

Data from this experiment were processed using SPSS Version 26.0 (IBM Corporation, Chicago, IL, United States). One-way ANOVA and Independent samples *t*-test were used to determine the significance of the data. The pan was considered as the experimental unit. In microbiological analysis, the alpha diversity index of intestinal microbes was analyzed using Tukey HSD. The VIP value of multivariate statistical analysis OPLS-DA and the *p*-value of univariate statistical analysis *t*-test were used to screen differential metabolites. Results are expressed as mean ± SEM, differences were considered significant at *p* < 0.05 (∗*p* < 0.05; ∗∗*p* < 0.01), and 0.05 < *p* < 0.10 was considered a tendency.

## 3 Results

### 3.1 Morphology analysis and nutrient quality of enzymatic pre-digestion diets

**Figure 1:**
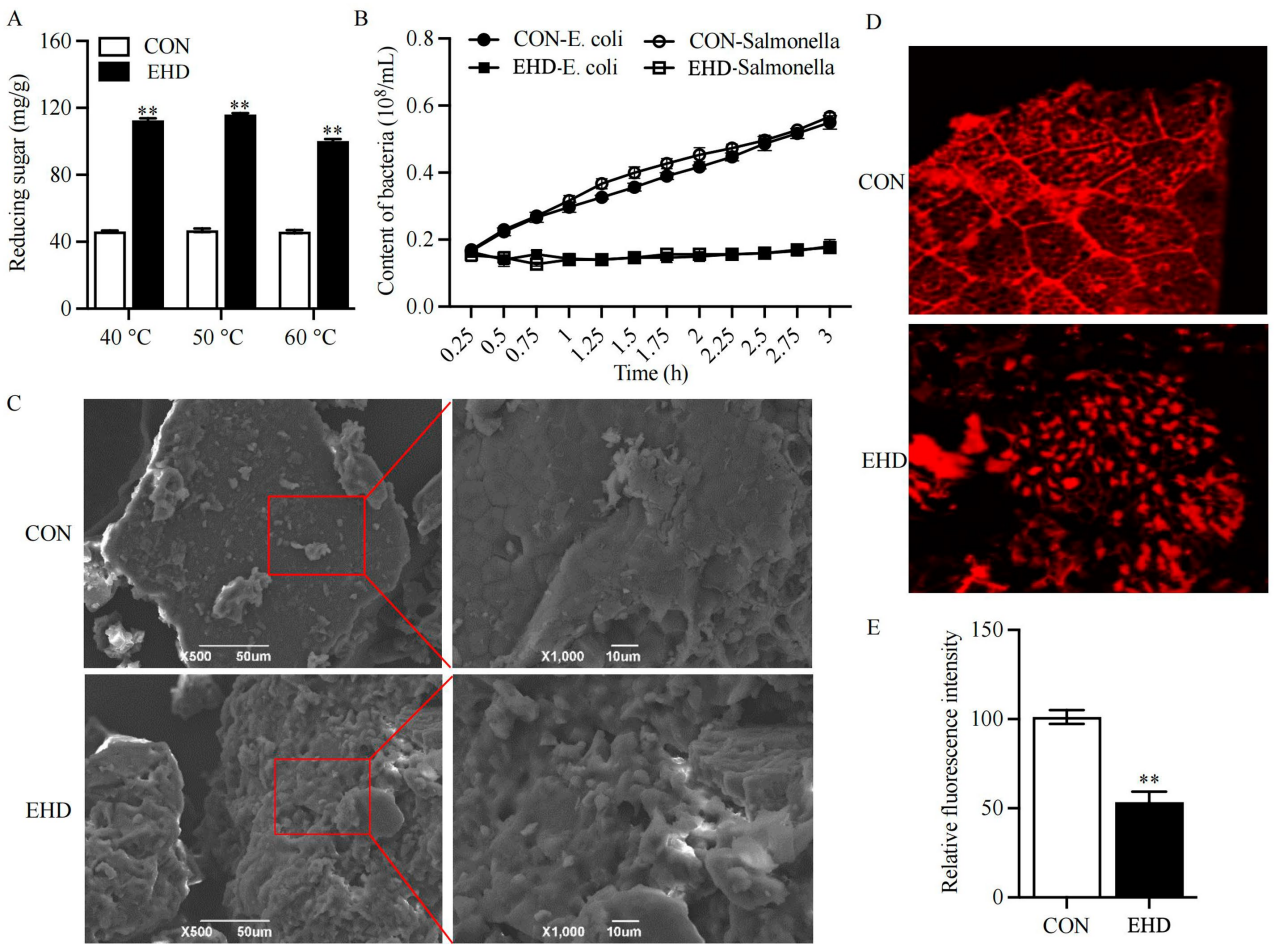
Reducing sugar content, antibacterial activity, and microstructure images of EHD. **(A)** Reducing sugar content at 40, 50, and 60°C. **(B)** Antagonistic activity of EHD against pathogens. **(C)** SEM images of diets at ×500 and ×1,000 magnification. **(D,E)** Laser confocal images and relative fluorescence intensity at ×100 magnification (mean ± SEM; *n* = 3 per group, *p < 0.05, **p < 0.01).

The complex enzyme pre-digestion treatment increased the reducing sugar content of the diets (Figure 1A) (*P* < 0.01). Given that there was no significant difference in reducing sugar content at the three temperatures tested, 40°C was selected as the enzymatic hydrolysis temperature for this experiment. Figure 1C illustrates the differences in surface structure between the basal (L-CON and S-CON) and EHD diets. The EHD surface showed more fragmented structures and larger pores compared to the smooth and flat basal diet. Additionally, the relative intensity of fluorescence on the EHD surface was reduced (Figures 1D,E) (*P* < 0.01).

The nutritional value of the experimental EHD is summarized in Table 4. OM and CP levels increased in the EHD group following enzymatic degradation (*p* < 0.01), while the EE, NDF, ADF, and ANFs (including Glycinin, *α*-conglycinin, *β*-conglycinin, and STI) levels decreased (*p* < 0.01). The total amino acids (TAA) concentration also rose under these conditions (*p* < 0.01). The gluconic acid content in the EHD group was 1.59 ± 0.03 μg/g; no gluconic acid was detected in the control groups. Furthermore, the EHD showed a lower pH value (Table 4) (*p* < 0.01) and inhibited the growth of pathogenic bacteria in the feed (Figure 1B).

**Table 4:** Effect of enzymatic pre-digestion treatment on the nutrient composition of diets.

Item	Diets^1^		*P*-value
	CON	EHD	
OM, %	80.01 ± 0.05^B^	84.35 ± 0.05^A^	< 0.001
CP, %	18.00 ± 0.07^B^	26.45 ± 0.06^A^	< 0.001
EE, %	15.56 ± 0.10^A^	8.47 ± 0.03^B^	< 0.001
NDF, %	11.19 ± 0.05^A^	9.67 ± 0.04^B^	< 0.001
ADF, %	4.43 ± 0.02^A^	3.86 ± 0.05^B^	0.001
TAA, μmol/g	0.024 ± 0.00^B^	0.018 ± 0.00^A^	< 0.001
α-conglycinin, μg/g	33.18 ± 0.84^A^	21.01 ± 1.96^B^	0.005
β-conglycinin, μg/g	49.25 ± 0.90^A^	25.72 ± 0.36^B^	< 0.001
Glycinin, μg/g	197.39 ± 7.29^A^	129.57 ± 2.19^B^	0.001
STI, ng/kg	216.61 ± 2.84^A^	107.07 ± 9.56^B^	< 0.001
pH	7.10 ± 0.03^A^	4.52 ± 0.02^B^	< 0.001
Gluconic acid, μg/g	-	1.59 ± 0.03	

OM, organic matter; CP, crude protein; EE, ether extract; NDF, neutral detergent fiber; ADF, acid detergent fiber; TAA, total amino acids; STI, soybean trypsin inhibitor. ^A,B^Different superscripts in each row for each factor differ significantly (*p* < 0.01). Data are presented as the mean ± SEM (*n* = 3).

1 CON, basal diet; EHD, enzymatically hydrolyzed diet.

### 3.2 Growth performance and serum biochemical indices

Table 5 shows that the EHD group exhibited an increase in average daily gain (ADG) (*p* < 0.01) and a decrease in the feed-to-gain ratio (F/G) (*p* < 0.01). However, there were no differences in initial and final BW and average daily feed intake (ADFI) among the three groups.

**Table 5:** Effects of EHD on growth performance of growing pigs.

Item	S-CON	L-CON	EHD	*P*-value
Initial weight, kg	27.69 ± 0.18	27.41 ± 0.44	27.25 ± 0.31	0.645
Final weight, kg	45.49 ± 0.34	45.54 ± 0.58	46.54 ± 0.52	0.305
ADG, g/d	658.72 ± 11.65^B^	671.33 ± 9.26^B^	714.67 ± 14.14^A^	0.003
ADFI, g/d	1745.52 ± 16.44	1707.87 ± 6.82	1677.47 ± 22.73	0.073
F/G	2.65 ± 0.02^Aa^	2.54 ± 0.01^Ab^	2.35 ± 0.03^Bc^	< 0.001

EHD, enzymatically hydrolyzed diet; S-CON, solid-control group; L-CON, liquid-control group; ADG, average daily gain; ADFI, average daily feed intake. ^a,b,c^Different superscripts in each row for each factor differ significantly (*p* < 0.05). ^A,B,C^Different superscripts in each row for each factor differ extremely significantly (*p* < 0.01). Data are presented as the mean ± SEM (*n* = 3).

Table 6 indicates that feeding pigs with EHD did not affect serum glucose levels. However, the concentrations of blood urea nitrogen (BUN) (*p* < 0.01) and total cholesterol (TCHO, *p* < 0.05) were lower in the EHD group. Additionally, while low-density lipoprotein cholesterol (LDL-C) levels remained unchanged, the EHD group showed increases in serum TP (*p* < 0.01), ALB (*p* < 0.05), GLB (*p* < 0.05), triglycerides (*p* < 0.05), and high-density lipoprotein cholesterol (HDL-C) (*p* < 0.05).

**Table 6:** Effects of EHD on serum biochemical indices of growing pigs.

Item	S-CON	L-CON	EHD	*P*-value
BUN, mmol/L	4.65 ± 0.14^A^	4.33 ± 0.06^A^	3.59 ± 0.10^B^	0.001
GLU, mmol/L	6.85 ± 0.14	6.86 ± 0.14	6.94 ± 0.04	0.859
TP, g/L	68.03 ± 0.57^B^	66.70 ± 1.27^B^	76.40 ± 0.70^A^	0.001
ALB, g/L	36.57 ± 0.69^b^	36.93 ± 1.18^b^	40.33 ± 0.44^a^	0.034
GLB, g/L	31.47 ± 0.43^ab^	29.77 ± 2.12^b^	36.07 ± 1.10^a^	0.045
TCHO, mmol/L	2.85 ± 0.07^A^	2.78 ± 0.03^A^	2.45 ± 0.07^B^	0.005
TG, mmol/L	0.78 ± 0.04^b^	0.78 ± 0.04^b^	0.97 ± 0.05^a^	0.033
HDL-C, mmol/L	0.93 ± 0.05^b^	0.98 ± 0.03^b^	1.12 ± 0.04^a^	0.030
LDL-C, mmol/L	0.98 ± 0.05	0.99 ± 0.02	0.96 ± 0.08	0.897

EHD, enzymatically hydrolyzed diet; S-CON, solid-control group; L-CON, liquid-control group; BUN, blood urea nitrogen; GLU, glucose; TP, total protein; ALB, albumin; GLB, globulin; TCHO, total cholesterol; TG, triglyceride; HDL-C, high-density lipoprotein cholesterol; LDL-C, low-density lipoprotein cholesterol. ^a,b,c^Different superscripts in each row for each factor differ significantly (*p* < 0.05). ^A,B,C^Different superscripts in each row for each factor differ extremely significantly (*p* < 0.01). Data are presented as the mean ± SEM (*n* = 3).

### 3.3 EHD improved the meat quality of growing pigs

Table 7 reveals no significant differences in pH at 45 m, pH at 24 h, and meat color L* value between different groups. However, pigs fed with EHD showed an increase in meat color a* value (*p* < 0.05) and a decrease in meat color b* value (*p* < 0.01). Moreover, EHD reduced both meat drip loss and shear force (*p* < 0.01).

**Table 7:** Effects of EHD on meat quality of growing pigs.

Item	S-CON	L-CON	EHD	*P*-value
Shear force, *N*	43.99 ± 0.18^A^	43.07 ± 0.16^B^	41.05 ± 0.14^C^	< 0.001
Drip loss, %	6.20 ± 0.10^A^	5.70 ± 0.12^B^	4.93 ± 0.10^C^	< 0.001
pH				
45 min	6.45 ± 0.03	6.44 ± 0.02	6.47 ± 0.03	0.801
24 h	5.51 ± 0.04	5.52 ± 0.04	5.47 ± 0.02	0.501
Color				
Lightness, L	42.11 ± 0.14	42.04 ± 0.10	41.78 ± 0.12	0.164
Redness, a	3.93 ± 0.05^b^	3.97 ± 0.04^b^	4.12 ± 0.05^a^	0.029
Yellowness, b	12.31 ± 0.08^A^	11.84 ± 0.06^B^	11.06 ± 0.09^C^	< 0.001

EHD, enzymatically hydrolyzed diet; S-CON, solid-control group; L-CON, liquid-control group. ^a,b,c^Different superscripts in each row for each factor differ significantly (*p* < 0.05). ^A,B,C^Different superscripts in each row for each factor differ extremely significantly (*p* < 0.01). Data are presented as the mean ± SEM (*n* = 3).

### 3.4 Nutrient digestibility and intestinal digestive enzyme content

Table 8 shows that the EHD group improved the digestibility of OM by 2%, CP by 3.54%, EE by 4.55%, NDF by 4.76%, and ADF by 5.33% compared to the S-CON group (*p* < 0.01). Compared to the L-CON group, the EHD group also improved the digestibility of OM by 2.75%, CP by 2.63%, EE by 3.51%, NDF by 4.95%, and ADF by 4.34% (*p* < 0.01). Furthermore, while the EHD did not have a statistically effect on the content of proteases and lipase, it did increase the levels of amylases (*p* < 0.01) and cellulase (*p* < 0.05) compared to both the S-CON and L-CON groups.

**Table 8:** Effects of EHD on nutrient digestibility and digestive enzyme content in growing pigs.

Item	S-CON	L-CON	EHD	*P*-value
Nutrient digestibility^1^, %				
Organic matter	86.12 ± 0.01^B^	85.37 ± 0.17^B^	88.12 ± 0.08^A^	< 0.001
Crude protein	72.68 ± 0.03^B^	73.59 ± 0.14^B^	76.22 ± 0.58^A^	0.001
Ether extract	77.30 ± 0.13^B^	78.34 ± 0.22^B^	81.85 ± 0.57^A^	< 0.001
Neutral detergent fiber	55.63 ± 0.56^B^	55.43 ± 0.25^B^	60.39 ± 0.13^A^	< 0.001
Acid detergent fiber	47.35 ± 0.09^B^	48.34 ± 0.47^B^	52.68 ± 0.27^A^	< 0.001
Digestive enzyme content				
Amylases, ng/g	3.36 ± 0.084^B^	3.40 ± 0.11^B^	4.51 ± 0.14^A^	< 0.001
Cellulase, ng/g	0.20 ± 0.01^Bb^	0.21 ± 0.01^Ab^	0.25 ± 0.01^Aa^	0.007
Proteases, ng/g	12.63 ± 0.39	13.42 ± 0.87	14.42 ± 0.61	0.208
Lipase, ng/g	0.22 ± 0.02	0.22 ± 0.01	0.27 ± 0.01	0.089

EHD, enzymatically hydrolyzed diet; S-CON, solid-control group; L-CON, liquid-control group. ^a,b,c^Different superscripts in each row for each factor differ significantly (*p* < 0.05). ^A,B,C^Different superscripts in each row for each factor differ extremely significantly (*p* < 0.01). Data are presented as the mean ± SEM (*n* = 3).

1 As air-drying basis.

### 3.5 Intestinal morphology, TJ proteins, and intestine inflammatory cytokines

**Figure 2:**
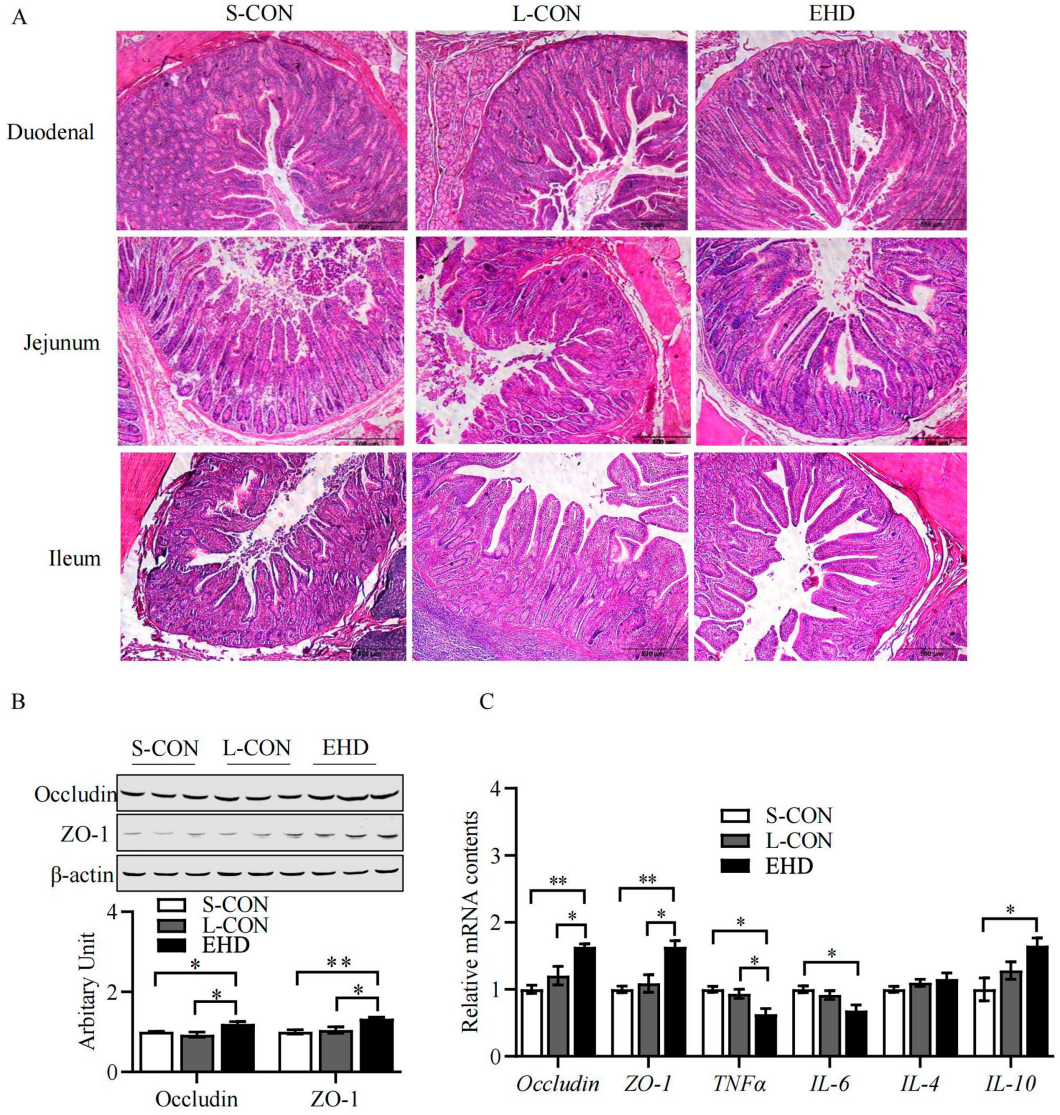
EHD improves intestinal morphology and mucosal barrier function. **(A)** H&E stained histology from S-CON, L-CON and EHD groups. **(B,C)** Expression of TJ proteins and inflammatory factors in the intestinal tract (mean ± SEM; *n* = 3 per group, *p < 0.05, **p < 0.01).

EHD improved intestinal development, as shown in Figure 2A. The villi were more uniformly arranged across all segments of the small intestine in the EHD group, with noticeable improvements in villi height, crypt depth, and villi-to-crypt ratios. These results are quantified using Image-J measurements and presented in Table 9. Villi height in the duodenum (*p* < 0.01), jejunum (*p* < 0.01), and ileum (*p* < 0.05) were greater in the EHD group compared to both S-CON and L-CON groups. The villi-to-crypt ratio was also higher in all small intestine segments in the EHD group than in the S-CON group (*p* < 0.01), with the ileum showing an increase compared to the L-CON group (*p* < 0.01).

**Table 9:** Effects of EHD on small intestinal morphology of growing pigs.

Item		S-CON	L-CON	EHD	*P*-value
Duodenum	Villus height, μm	869.80 ± 10.03^C^	954.36 ± 10.26^B^	1084.14 ± 13.12^A^	< 0.001
	Crypt depth, μm	483.49 ± 6.29^Bb^	492.10 ± 3.67^ABb^	516.48 ± 3.63^Aa^	0.006
	VH/CD	1.80 ± 0.04^Bc^	1.94 ± 0.01^ABb^	2.1 ± 0.03^Aa^	0.002
Jejunum	Villus height, μm	788.49 ± 9.97^C^	881.37 ± 12.51^B^	957.08 ± 11.38^A^	< 0.001
	Crypt depth, μm	474.83 ± 7.20	473.69 ± 10.40	479.14 ± 9.66	0.908
	VH/CD	1.66 ± 0.02B^c^	1.86 ± 0.03^Ab^	2.00 ± 0.04^Aa^	< 0.001
Ileum	Villus height, μm	784.72 ± 19.83^b^	795.99 ± 19.42^b^	861.18 ± 8.36^a^	0.036
	Crypt depth, μm	474.53 ± 14.33^ab^	494.24 ± 9.75^a^	439.42 ± 9.50^b^	0.038
	VH/CD	1.65 ± 0.01B	1.61 ± 0.01^B^	1.96 ± 0.03^A^	< 0.001

EHD, enzymatically hydrolyzed diet; S-CON, solid-control group; L-CON, liquid-control group; V/C, Villus height/Crypt depth. ^a,b,c^Different superscripts in each row for each factor differ significantly (*p* < 0.05). ^A,B,C^Different superscripts in each row for each factor differ extremely significantly (*p* < 0.01). Data are presented as the mean ± SEM (*n* = 3).

The abundance of TJ proteins Occludin and ZO-1 was elevated in the EHD group (Figures 2B,C) (*P* < 0.05). Additionally, pigs fed with EHD exhibited lower mRNA levels of the pro-inflammatory cytokines TNF-*α* and IL-6 (Figure 2C) (*P* < 0.05). Conversely, the mRNA level of the anti-inflammatory cytokine IL-10 was higher in the EHD group (*p* < 0.05).

### 3.6 Colonic SCFAs

The concentrations of acetate, propionate, and butyrate in the colon were higher in the EHD group than in both control groups (Table 10) (*p* < 0.01). Similarly, the isovalerate level was higher in the EHD group compared to the S-CON (*p* < 0.01) and L-CON (*p* < 0.05) groups. However, the valerate and isobutyrate contents remained unchanged. Between the two control groups, the L-CON group showed elevated levels of acetate (*p* < 0.01), propionate (*p* < 0.05), and butyrate (*p* < 0.05).

**Table 10:** Effects of EHD on short chain fatty acids levels in colon chyme of growing pigs.

Item	S-CON	L-CON	EHD	*P*-value
Acetate, mol/g	2.67 ± 0.10^C^	3.13 ± 0.01^B^	3.54 ± 0.02^A^	0.001
Propionate, mol/g	1.12 ± 0.03^Bc^	1.34 ± 0.07^Bb^	1.72 ± 0.03^Aa^	0.001
Butyrate, mol/g	0.58 ± 0.01^Bc^	0.83 ± 0.03^Bb^	1.11 ± 0.04^Aa^	0.001
Isobutyrate, mol/g	0.025 ± 0.0001	0.024 ± 0.0002	0.025 ± 0.0009	0.892
Isovalerate, mol/g	0.08 ± 0.01^Bb^	0.14 ± 0.04^ABb^	0.36 ± 0.03^Aa^	0.013
Valerate, mol/g	0.138 ± 0.003	0.141 ± 0.004	0.140 ± 0.003	0.834

EHD, enzymatically hydrolyzed diet; S-CON, solid-control group; L-CON, liquid-control group. ^a,b,c^Different superscripts in each row for each factor differ significantly (*p* < 0.05). ^A,B,C^Different superscripts in each row for each factor differ extremely significantly (*p* < 0.01). Data are presented as the mean ± SEM (*n* = 3).

### 3.7 Fecal microbiota

**Figure 3:**
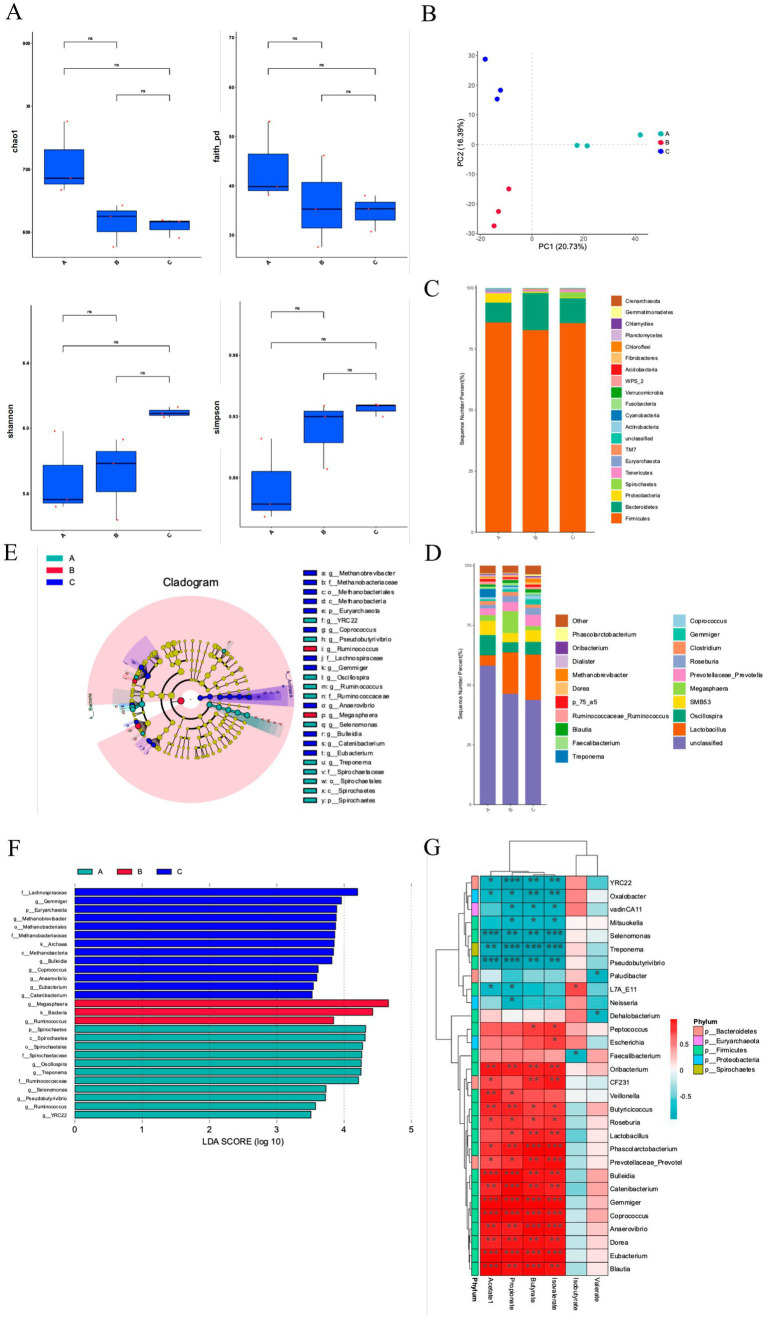
EHD alters colonic microbiota in growing pigs. **(A)** Fecal microbiota alpha diversity, including Chao index, Faith index, Shannon index, and Simpson index analysis results. **(B)** Principal component analysis of fecal microbiota. **(C,D)** The relative abundance of fecal flora at the phylum and genus level. **(E)** LEfSe multi-level species tree map. **(F)** LDA discriminant result map. **(G)** Correlation analysis between fecal flora and short-chain fatty acids. EHD = enzymatic predigested diet; **(A)** = group S-CON; **(B)** = group L-CON; **(C)** = group EHD. (mean ± SEM; *n* = 3 in each group).

Four indices (Chao 1, Faith, Shannon, and Simpson 4) were used to assess the α-diversity of fecal microorganisms (Figure 3A). Although there were no significant differences among the three groups, the EHD group exhibited a trend-level decrease in Chao 1 and a trend-level increase in Shannon. Principal component analysis indicated a distinct separation of microbiota clusters among the three groups (Figure 3B), with the first and second principal components accounting for 20.73 and 16.39% of the variance, respectively.

At the phylum level, the dominant flora included *Firmicutes*, *Bacteroidetes*, and *Proteobacteria* (Figure 3C). *Firmicutes* was the predominant phylum, comprising 85.89, 82.73, and 85.57% of the microbiota in the S-CON, L-CON, and EHD groups, respectively, with no significant differences among the groups. Interestingly, the proportion of *Bacteroidetes* was higher in the L-CON group compared to the S-CON group (*p* < 0.01) and the EHD group (*p* < 0.05). Conversely, *Proteobacteria* levels were higher in L-CON group compared to the L-CON and EHD groups (*p* < 0.01).

At the genus level, 20 bacterial genera with relatively high abundance were identified (Figure 3D). *Lactobacillus* emerged as the dominant genus. The relative abundances of *Lactobacillus*, *Prevotella*, *Coprococcus*, and *Blautia* were 19.04, 4.74, 1.59, and 1.33%, respectively, all higher than in the S-CON group (*p* < 0.01). *Oscillospira* and *Roseburia* were lower in the EHD group compared to the S-CON group, with relative abundances of 5.29 and 4.90%, respectively (*p* < 0.01). Additionally, *Megasphaera* was lower in the EHD group compared to the L-CON group, accounting for 1.73% (*p* < 0.01). *Phascolarctobacterium* was higher in the EHD group compared to both control groups, accounting for 0.74% (*p* < 0.01).

Further analysis using a multi-level species tree and applying linear discriminant analysis (LDA) and LEfSe analysis revealed 27 different fecal bacterial genera among the three groups (Figures 3E,F) (*P* < 0.05). Notably, *Lactobacillus*, *Gemmatobacteria*, and *Methanobacterium* showed differences in the EHD group (*p* < 0.05). Interestingly, the different flora between the control groups varied, with *Megasphaera* and *Ruminococcus* being prominent in the L-CON group (*p* < 0.05) and *Spirochaetes*, *Spirochaetale*, and *Oscillospira* in the S-CON group (*p* < 0.05).

### 3.8 Relationship of the fecal microbiota community with colonic SCFAs

To explain the impacts of EHD on the gut microbiota of growing pigs, we analyzed the correlations of the significantly different SCFA content with the fecal microbiota (Figure 3G). *Lactobacillus* was positively correlated with Propionate, Butyrate, and Isobutyrate (*p* < 0.05). Acetate, propionate, butyrate, and isobutyrate were positively correlated with *Phascolarctobacterium*, *Coprococcus*, and *Prevotella* (*p* < 0.05) and negatively correlated with Pseudobutyrivibrio and Selenomonas (*p* < 0.05). Valerate and isovalerate were not significantly associated with most bacterial groups.

## 4 Discussion

Previous studies have indicated that enzymatic pretreatment of soybean meal enhances feed bioavailability (29) and improves swine growth performance (30), but most of these studies focus on pretreating raw materials and produce inconsistent results (15). This study established an *in vitro* enzyme pre-digestion system and evaluated its quality as corn-soybean meal EHD. The findings demonstrate that enzyme pre-digestion increased dietary bioavailability and antimicrobial activity, and pigs fed with EHD exhibited improved growth performance, meat quality, and intestinal health.

In this experiment, the bioavailability of EHD was significantly increased, primarily through the significant reductions in ANFs and increases in nutrients. The plant ingredients in a corn-soybean meal diet interact to form insoluble complexes that hinder nutrient release, with ANFs being the primary cause (31). This study found that levels of *α*-conglycinin, *β*-conglycinin, Glycinin, and STI were significantly reduced after enzymatic pre-digestion, with β-conglycinin and STI decreasing by 47.78 and 50.57%, respectively. This indicates that the compound enzyme preparation used in this experiment was highly effective in reducing ANFs, in concordance with previous experimental data (32). Additionally, soluble NSP in the diet increases chyme viscosity, leading to intestinal edema (33, 34). In this study, the significant reduction in the sugar content of the EHD after pre-digestion serves as an indicator of NSP degradation (35). This is because enzymatic treatment can specifically and rapidly degrade NSPs into reducing sugars (36). Moreover, the disruption of cell wall structure on the surface of EHD feed is likely due to the degradation of NSP enzymes, consistent with the enzyme-induced disruption of cell wall structure by *Bacillus subtilis* (37).

The nutritional levels and quality of the EHD were also improved. The enzyme preparation used in this study included digestive enzymes beyond just those targeting NSPs. The increase in TAA content indicates that larger protein molecules were converted into smaller, more easily absorbed molecules, improving the utilization of feed OM and CP (38). The presence of lipase in EHD significantly reduced the EE content. Additionally, it has been demonstrated that exogenous cellulases, hemicellulases, and proteases can similarly lower the ADF and NDF content (39). Furthermore, the gluconic acid produced by the enzymatic digestion of glucose oxidase created a low pH acidic environment (40), inhibiting the growth of *Escherichia coli* and *Salmonella* in the feed (41). The addition of lysozyme may have destroyed the cell walls of harmful bacteria, consistent with previous findings (42).

Significant improvements in the growth performance of growing pigs are closely linked to increased nutrient digestibility in the diet (43, 44). In this experiment, there were significant changes in the average daily weight gain and feed-to-weight ratio of growing pigs. Nutrient digestibility was significantly improved for several nutrients, including OM, CP, EE, NDF, and ADF.

Blood biochemical indicators are frequently used to assess the nutritional and health status of growing pigs. In this experiment, TP, ALB, and BUN were key indicators of protein metabolism. Lower BUN values indicated efficient amino acid metabolism in the diet (45), while higher TP and ALB levels suggested improved protein synthesis, likely due to enzyme pretreatment of the feed, which reduced chyme blockage caused by NSP and promoted protein absorption. Serum triglyceride levels, an important intermediate in lipid metabolism derived from feed or liver synthesis, were found to increase with enzyme supplementation, consistent with the findings of Shanti et al. (46). The increase in serum HDL-C content and the decrease in cholesterol levels indicated improved lipid metabolism. Additionally, the lower serum cholesterol could be attributed to changes in intestinal flora structure due to EHD, with a significant increase in SCFAs, particularly propionic acid produced by lactic acid bacteria, limiting hepatic cholesterol synthesis, as observed in previous studies (47).

There exist several interrelated meat quality indicators, with meat redness primarily associated with myoglobin content. Some studies have shown that enzyme supplementation in broiler feed does not significantly affect meat quality (48, 49). However, in this experiment, meat redness increased in the EHD group, possibly due to species differences. Drip loss and cooking loss were significantly lower in the EHD group compared to the control groups. Previous studies have indicated that lower water loss and cooking loss help retain more meat juices during thawing and processing, which is crucial for evaluating meat quality (50). Additionally, drip loss in pork was correlated with the rate of pH decrease (pH_45min_, pH_24h_).

Intestinal digestion and absorption in pigs are directly related to their growth performance (44). An increase in intestinal digestive enzymes improves nutrient digestibility (51). Among feed ANFs, *β*-conglycinin has been noted to cause significant intestinal immunogenic reactions (52). In this experiment, EHD significantly reduced dietary ANFs, minimized the damage caused by antigenic proteins to intestinal villi and mucosa, and improved intestinal structure by arranging villi more uniformly compared to the control group. Nutrient uptake in the small intestine depends largely on the integrity and morphology of the intestinal villi. The greater the height and the higher the villus-to-crypt ratio, the larger the absorption surface area, increasing intestinal absorption capacity (53).

Additionally, improvements in the mRNA abundance of TJ proteins in the ileum are likely due to the reduced concentrations of glycinin and β-conglycinin in enzymatically treated soybean meal (15, 44). In the present study, the EHD group showed up-regulation of TJ expression and down-regulation of pro-inflammatory factors in the colon. The intestinal epithelial barrier functions as a defense against pathogen transit from the intestinal lumen into the body (15). The TJ barrier in the intestinal epithelium regulates the paracellular permeation of intestinal contents into the mucosa and somatic circulation. Recent studies indicate that intestinal inflammatory factors, including TNF-*α*, IL-1β, and IL-*γ*, play significant roles in regulating the intestinal TJ barrier (54).

Feeds for monogastric animals like pigs often contain carbohydrates that are not fully digested in the small intestine and instead reach fermenting microorganisms in the posterior segment of the intestine, where they produce SCFAs (55). These SCFAs are the final metabolites of dietary fiber and protein, mainly produced in the colon (56). In this experiment, the levels of acetate, propionate, and butyrate were significantly higher in the colons of pigs fed with EHD. The NSPs and ANFs present in feed can hinder proper nutrient breakdown and can lead to the production of harmful fermentation byproducts in the colon (57).

Butyric acid, a component of SCFAs, provides energy to intestinal epithelial cells, aids in repairing the intestinal mucosal barrier, and supports the immune system. It has been shown to supply 15% of the energy requirements for growing pigs (58). Additionally, the increase in the relative abundance of lactic acid bacteria leads to the production of more SCFAs (59), particularly propionic acid (47). The production of these SCFAs also helps maintain an anaerobic environment in the colon, which inhibits the growth of harmful bacteria such as *Salmonella* (60).

The animal intestine hosts a rich microbiota, with microbial activity significantly affecting the host’s nutritional metabolism and physiological functions (61). Studies have shown that adding enzymes to feed can regulate intestinal flora structure. Studies have demonstrated that glucose oxidase in feed improved the intestinal microbial composition of broiler chickens by consuming oxygen during enzyme degradation to produce gluconic acid (62). This process increased the relative abundance of *Firmicutes*, which were the dominant phylum in this study, consistent with previous findings (61).

In this experiment, a decrease in the Chao1 index indicated that EHD reduced the number of intestinal flora in growing pigs, while an increase in the Shannon index suggested a greater diversity of intestinal flora (62). This may be due to the enzymatic treatment inhibiting the growth of harmful bacteria. Changes in intestinal microorganisms also affected SCFAs, showing significant correlations between them (63). Enrichment of probiotic flora can induce competitive rejection of pathogenic bacteria by occupying binding sites in the intestinal mucosa or by competing with pathogenic bacteria for nutrient and absorption sites (64). Lactobacilli are receiving increasing attention as probiotics in swine due to their ability to improve growth performance and carcass quality, prevent gastrointestinal infections, and, most importantly, their “recognized safety” properties. Lactobacilli can adhere to the intestinal epithelium and thus compete with pathogens for adhesion receptors (65). Methanobacterium is an archaeon present in the intestinal microbiota of swine and has a significant impact on gut health. They promote gut health and function by participating in nutrient metabolism, modulating the immune system, and providing resistance to pathogens, particularly including management of digestive energy utilization and methane emissions (66).

Further, the results of this study found that various bacterial genera, such as *Lactobacillus*, showed a significant positive correlation with propionic acid, butyric acid, and isobutyric acid. *Lactobacillus* improves intestinal barrier function, balances intestinal flora, regulates the immune system, and increases resistance to pathogenic bacteria (67, 68). *Prevotella* is an important component of the *Bacteroidetes* and promotes the digestion and absorption of cellulose in the animal body (69). *Blautia* improves immune function and reduces cholesterol levels (70). Moreover, there was a significant correlation between beneficial genera such as *Lactobacillus*, *Prevotella*, and *Coprococcus* and SCFAs in the EHD group, indicating that EHD improved the digestion and utilization of cellulose. *Coprococcus* can break down fructose, lactose, and galactose (71). In this experiment, the content of *Caldicoprobacter faecalis* in pigs fed EHD was significantly higher than in the control groups, likely because EHD increased the reducing sugar content in the feed, stimulating the proliferation of *Caldicoprobacter faecalis*. Therefore, we believe that following a month-long adaptation of the EHD diet, it ultimately engendered significant alterations and enhancements in the overall impact on the gut microbiota of growing pigs. However, the longitudinal changes of microbiota during this process also deserve to be scrutinized and discussed, contributing to a more realistic and accurate understanding of the dynamic changes produced by EHDs on gut microbiota, and is a key focus of our future research.

## 5 Conclusion

In summary, enzymatic pre-digestion of diet can reduce dietary atrial natriuretic factors (ANFs), increase the apparent digestibility of feed and the content of intestinal digestive enzymes in growing pigs, improve the intestinal structure and the distribution of intestinal microbial flora, and increase the proportion of beneficial bacteria. These factors contribute to improving the intestinal health of growing pigs.

## Data Availability

The datasets presented in this study can be found in online repositories. The names of the repository/repositories and accession number(s) can be found in the article/supplementary material.

## Footnotes

^1^ https://www.bioincloud.tech
